# Erythropoiesis and Gene Expression Analysis in Erythroid Progenitor Cells Derived from Patients with Hemoglobin H/Constant Spring Disease

**DOI:** 10.3390/ijms252011246

**Published:** 2024-10-19

**Authors:** Narawich Wongkhammul, Pinyaphat Khamphikham, Siripong Tongjai, Adisak Tantiworawit, Kanda Fanhchaksai, Somsakul Pop Wongpalee, Alisa Tubsuwan, Supawadee Maneekesorn, Pimlak Charoenkwan

**Affiliations:** 1 Center of Multidisciplinary of Advanced Medicine, Faculty of Medicine, Chiang Mai University, Chiang Mai 50200, Thailand; narawich_wo@cmu.ac.th; 2 Department of Biochemistry, Faculty of Medicine, Chiang Mai University, Chiang Mai 50200, Thailand; 3 Division of Clinical Microscopy, Department of Medical Technology, Faculty of Associated Medical Sciences, Chiang Mai University, Chiang Mai 50200, Thailand; pinyaphat.kha@cmu.ac.th; 4 Hematology and Health Technology Research Center, Department of Medical Technology, Faculty of Associated Medical Sciences, Chiang Mai University, Chiang Mai 50200, Thailand; 5 Department of Microbiology, Faculty of Medicine, Chiang Mai University, Chiang Mai 50200, Thailand; siripong.tongjai@cmu.ac.th (S.T.); somsakul.w@cmu.ac.th (S.P.W.); 6 Division of Hematology, Department of Internal Medicine, Faculty of Medicine, Chiang Mai University, Chiang Mai 50200, Thailand; adisak.tan@cmu.ac.th; 7 Thalassemia and Hematology Center, Faculty of Medicine, Chiang Mai University, Chiang Mai 50200, Thailand; iamkanda54@gmail.com (K.F.); supawadee.man@cmu.ac.th (S.M.); 8 Division of Hematology and Oncology, Department of Pediatrics, Faculty of Medicine, Chiang Mai University, Chiang Mai 50200, Thailand; 9 Institute of Molecular Biosciences, Mahidol University, Nakhon Pathom 73170, Thailand; alisa.tub@mahidol.ac.th

**Keywords:** α-thalassemia, erythropoiesis, gene expression, hemoglobin H/Constant Spring disease, molecular chaperone

## Abstract

Hemoglobin H/Constant Spring (Hb H/CS) disease represents a form of non-deletional Hb H disease characterized by chronic hemolytic anemia that ranges from moderate to severe and may lead to transfusion-dependent thalassemia. To study the underlying mechanisms of this disease, we conducted an analysis of erythropoiesis and gene expression in erythroid progenitor cells derived from CD34+ hematopoietic stem/progenitor cells from patients with Hb H/CS disease and normal controls. Twelve patients with Hb H/CS disease and five normal controls were enrolled. Peripheral blood samples were collected to isolate CD34+ hematopoietic stem/progenitor cells for the analysis of cell proliferation and differentiation. Six samples from patients with Hb H/CS disease and three controls were subsequently studied for gene expression by next generation sequencing analysis. Erythroid progenitor cells derived from patients with Hb H/CS disease exhibited a trend towards increased rates of erythroid proliferation and decreased cell viability compared to those from controls. Moreover, erythroid progenitor cells derived from patients with Hb H/CS disease demonstrated delayed terminal differentiation. Gene expression profiling revealed elevated levels of genes encoding molecular chaperones, including the heat shock protein genes (*HSP*s) and the chaperonin containing TCP-1 subunit genes (*CCT*s) in the Hb H/CS disease group. In summary, erythroid progenitor cells derived from patients with Hb H/CS disease exhibit a trend towards heightened erythroid proliferation, diminished cell viability, and delayed terminal differentiation. Additionally, the increased expression of genes encoding molecular chaperones was observed, providing information on potential underlying pathophysiological mechanisms.

## 1. Introduction

Hemoglobin H (Hb H) disease is the most prevalent form of thalassemia disease in Thailand, affecting approximately one in one hundred individuals [1,2,3]. Hb H disease arises from mutations in three out of the four functioning α-globin genes (*HBA*), resulting in decreased production of Hb A (α2β2) and the formation of abnormal Hbs, namely Hb H (β4) and Hb Bart’s (γ4). Hb H disease can be categorized into two subtypes: deletional Hb H disease, caused by deletions of three α-globin alleles, and non-deletional Hb H disease, resulting from in cis deletions of two α-globin alleles on one chromosome and a point mutation on the other chromosome. Patients with non-deletional Hb H disease typically experience more severe anemia compared to those with deletional Hb H disease [4,5].

Hb Constant Spring (Hb CS), resulting from the point mutation *HBA2*:c.427T>C, represents the most prevalent type of non-deletional mutation observed in *HBA* among populations in Southeast Asia [1,3,6]. This mutation leads to the substitution of the termination codon in the *HBA2* gene with glutamine, resulting in an elongated α-globin chain that includes an additional 31 amino acids [7]. The presence of α^CS^ can give rise to the formation of abnormal precipitates within red blood cells and can alter the structure of red blood cell membrane proteins [8]. Compound heterozygous mutations involving α^0^-thalassemia and Hb CS lead to the development of Hb H/CS disease. Patients diagnosed with Hb H/CS disease typically exhibit a moderate clinical severity of chronic hemolytic anemia, with some requiring long-term red blood cell transfusions [5,9].

While the majority of patients with Hb H/CS disease typically present with moderate, non-transfusion-dependent thalassemia, in certain instances, the disease can manifest as severe anemia. Hb H/CS disease may even lead to hydropic changes in the fetus, potentially resulting in fetal demise [10,11]. Moreover, severe fetal anemia and hydrops fetalis have been observed in association with homozygous Hb CS [12,13,14,15,16]. In selected cases, intrauterine transfusion has been demonstrated to mitigate further complications and render favorable outcomes [12,14,16]. However, the genetic modifiers or factors associated with the severity of Hb H/CS disease remain largely elusive. Most research on disease pathophysiology has focused on β-thalassemia, which exhibits distinct pathophysiological mechanisms compared to α-thalassemia [17,18,19,20,21]. This study aimed to investigate the process of erythropoiesis in individuals with Hb H/CS disease. The insights gained will lay a foundational framework for the advancement of treatments aimed at alleviating disease severity in affected individuals.

## 2. Results

Twelve patients diagnosed with Hb H/CS disease and five normal controls were included in the study. Demographic data and hematological characteristics are presented in Table 1. The patients were notably younger than the controls, while the sex distribution was similar between groups. Significant differences were observed in the red blood cell parameters between the two groups.

### 2.1. Cell Proliferation and Viability

**Figure 1:**
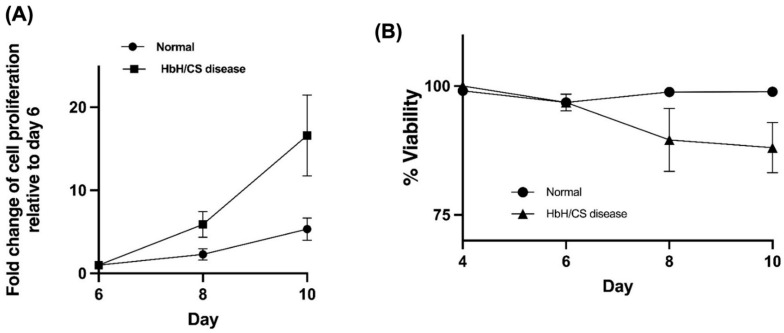
Comparison of cell proliferation (**A**) and viability (**B**) of cultured erythroid cells derived from patients with hemoglobin H/Constant Spring (Hb H/CS) disease (N = 12) and controls (N = 5). The fold change of cell proliferation represents the ratio of cell number at the indicated time points versus day 6. Data are presented as the mean ± standard error of the mean (SEM).

The assessment of cell proliferation, as measured by fold change in cell counts relative to day 6, indicated that erythroblasts derived from patients with Hb H/CS disease have a higher cell proliferation than those from controls on days 8 and 10 (Figure 1A). However, this difference did not reach statistical significance (*p*-value = 0.194). Additionally, an examination of cell viability on days 4, 6, 8, and 10 revealed a trend towards decreased cell viability among erythroblasts from patients with Hb H/CS disease. However, this difference was also not statistically significant (*p*-value = 0.436) (Figure 1B).

### 2.2. Cell Differentiation

**Figure 2:**
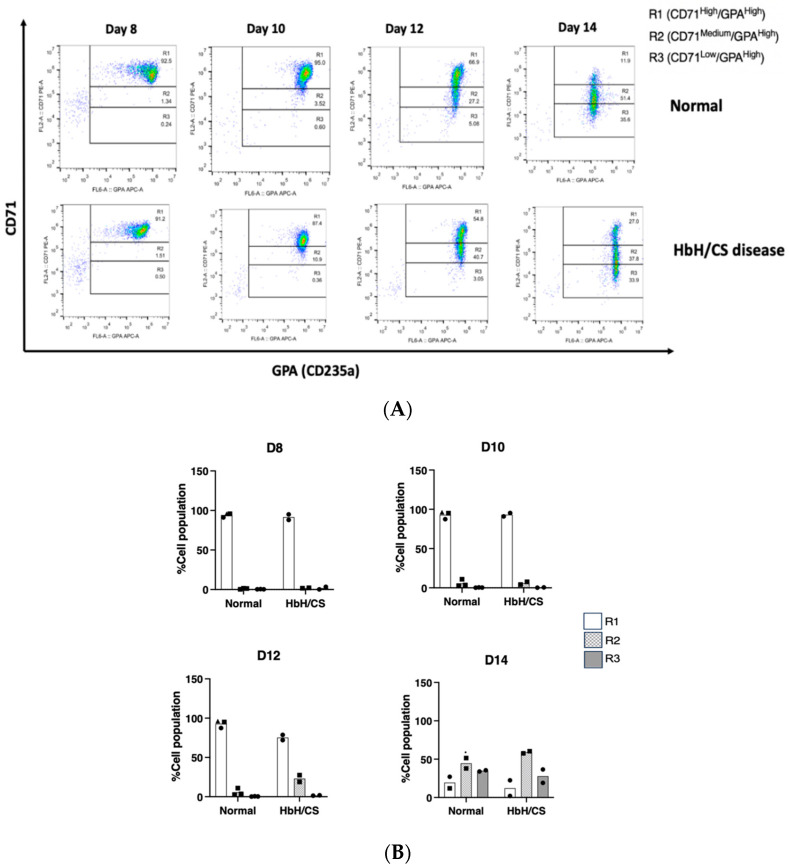
Cell differentiation studies in erythroblasts derived from patients with hemoglobin H/Constant Spring (Hb H/CS) disease and controls. Delayed erythroid differentiation is observed in Hb H/CS disease. (**A**) A representative flow cytometry dot plot for erythroid differentiation analysis during a three-phase ex vivo erythroid cell culture from a normal individual and a patient with Hb H/CS disease. (**B**) The histogram represents the quantitation of erythroid differentiation analyzed by flow cytometry at days 8, 10, 12, and 14 in normal controls (N = 3) and Hb H/CS disease (N = 2).

Cell differentiation studies were performed in two patients with Hb H/CS disease and three controls. The results are illustrated in Figure 2. On day 12, erythroid precursor cells derived from the patients progressed to the R2 gate more rapidly compared to those from the controls. However, by day 14, erythroid precursor cells from these patients were observed to accumulate in the R2 gate, indicating a potential arrest in differentiation during the later stages of erythropoiesis.

### 2.3. Gene Expression Analysis

**Figure 3:**
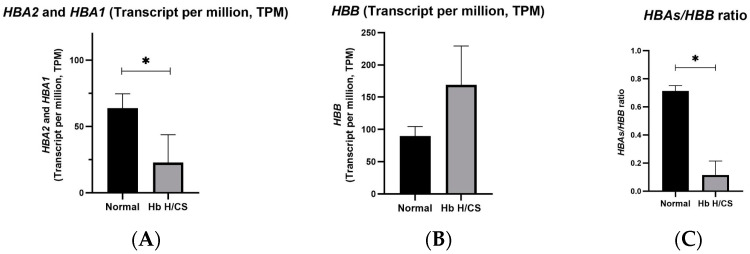
The expression of the genes of interest, *HBA*s and *HBB*, was represented using transcripts per million (TPM) values. (**A**) *HBA2* and *HBA1* mRNA expression in erythroid cells derived from normal controls (N = 3) and patients with hemoglobin H/Constant Spring (Hb H/CS) disease (N = 6) showed a significant difference (*p*-value = 0.0216). (**B**) The mRNA expression of *HBB* between normal controls and patients with Hb H/CS disease did not show a significant difference (*p*-value = 0.0735). (**C**) The *HBA*s/*HBB* ratio between normal controls and patients with Hb H/CS disease showed a significant difference (*p*-value < 0.0001). Statistical significance was determined using *t*-tests, with a *p*-value of <0.05 considered significant. * *p* < 0.05 when compared with normal controls.

The TPM values of *HBA2* and *HBA1* in erythroid precursor cells derived from the patients with Hb H/CS disease (N = 6) were significantly lower than those in the controls (N = 3) (*p*-value = 0.0216), indicating that the *HBA2* and *HBA1* gene expression was insufficient in Hb H/CS disease (Figure 3A). The *HBB* gene expression levels in erythroid precursor cells derived from the patients were higher than those in the controls. However, the difference was not statistically significant (*p*-value = 0.0738), which may be attributed to the small sample size (Figure 3B). The *HBA*s/*HBB* ratios in the patients were significantly lower than those in the controls (*p*-value < 0.0001) (Figure 3C), reflecting the imbalanced globin synthesis.

**Figure 4:**
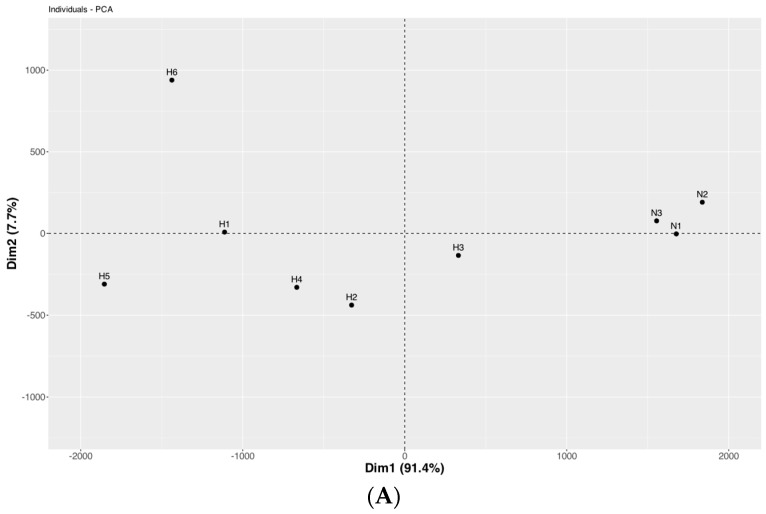

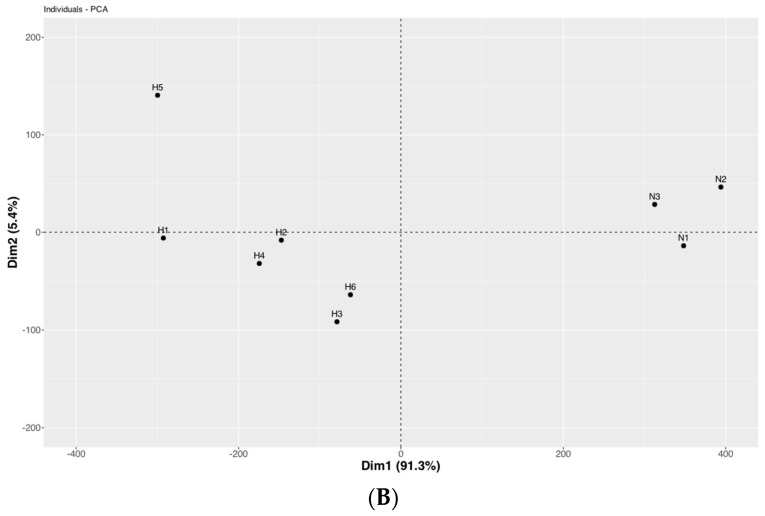
The Principal Component Analysis (PCA) gene expression datasets. N1–N3 represent normal controls and H1–H6 represent patients with hemoglobin H/CS disease. (**A**) The PCA of nine heat shock protein (*HSP*) gene expression datasets and (**B**) the PCA of nine chaperonins containing TCP-1 complex (*CCT*) gene expression datasets.

Principal Component Analysis (PCA) results on nine gene expression datasets revealed a distinct separation between a cluster of three control samples and the six samples from patients with Hb H/CS disease, as illustrated in Figure 4. Further analysis identified that the genes contributing significantly to this separation included heat shock protein genes (*HSP*s): *HSPA8*, *HSP90AA1*, *HSP90AB1*, *HSPA1A*, and *HSPA1B* (Figure 4A), and all nine chaperonin containing TCP-1 subunit genes (*CCT*s), including *TCP1*, *CCT2*, *CCT3*, *CCT4*, *CCT5*, *CCT6A*, *CCT6B*, *CCT7,* and *CCT8* (Figure 4B).

**Figure 5:**
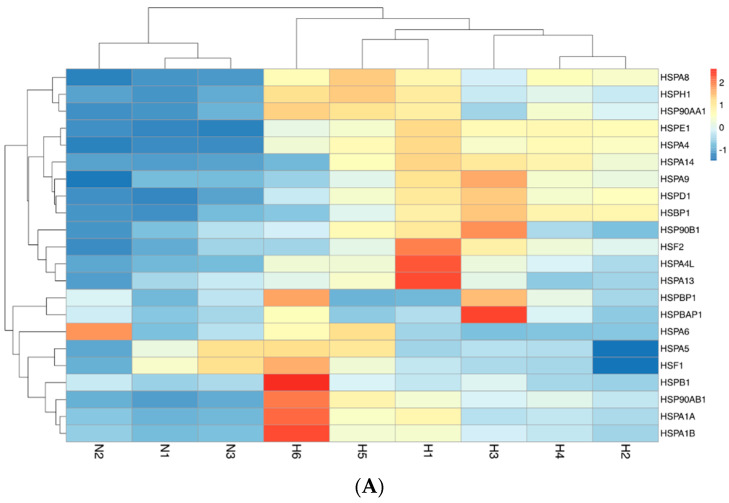

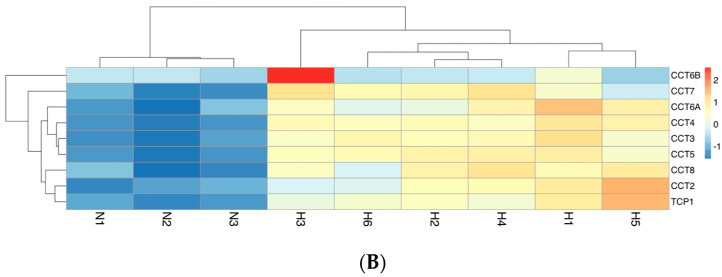
A heatmap of the gene expression. N1–N3 represents normal controls and H1–H6 represents patients with hemoglobin H/CS disease. A heatmap of the 22 heat shock protein (*HSP*) gene expression (**A**) and nine chaperonins containing TCP-1 complex (*CCT*) gene expression (**B**).

Additional gene expression pattern analysis supported the findings from the PCA. Figure 5A shows all normal samples clustered together, displaying lower expressions of *HSP* genes, with exceptions being relatively higher expressions of *HSPA6* in one control and elevated expressions of *HSPA5* and *HSF1* in two controls. Samples from the Hb H/CS disease group formed a distinct cluster. Notably, one Hb H/CS disease sample was placed in a unique clade, showing upregulation of *HSPB1*, *HSPA1A*, *HSPA1B*, *HSP90AB1*, and *HSF1*.

Gene expression analysis of the *CCT* gene family revealed a clear distinction between the control group and patients with Hb H/CS disease, with the two groups forming distinct clusters. All nine *CCT* genes exhibited significantly elevated expression levels in patients with Hb H/CS disease.

## 3. Discussion

The present study investigated erythropoiesis and gene expression in patients diagnosed with Hb H/CS disease compared to normal controls. Our findings revealed distinct differences in cell proliferation, viability, and differentiation between the two groups. In terms of cell proliferation, our results indicated a trend towards higher proliferation among erythroblasts from patients with Hb H/CS disease compared to controls. Although this difference did not reach statistical significance, it suggests a potential dysregulation in erythroid proliferation dynamics in Hb H/CS disease. Similarly, examination of cell viability revealed a trend towards decreased viability among erythroblasts from patients with Hb H/CS disease. Moreover, investigation of the differentiation process suggests a potential disruption or delay in erythroid maturation during the later stages of erythropoiesis in Hb H/CS disease.

Kaewsakulthong et al. found that all types of thalassemia demonstrated increased cell expansion, with β^0^-thalassemia/Hb E disease showing the highest level [22]. Erythroid cells from Hb H/CS patients showed greater expansion than those of controls. Our findings align with this previous study, indicating the ineffective erythropoiesis characteristics of Hb H/CS disease. However, this finding contrasts with a study by Sriiam et al., which suggested a reduced expansion of Hb H/CS disease erythroid precursors compared to normal controls [23]. Our study also observed delayed erythroid differentiation in Hb H/CS disease compared to controls, aligning with previous findings [22]. Conversely, Sriiam et al. found no difference in differentiation among Hb H/CS erythroblasts [23]. These findings highlight the complexity of erythropoietic dysregulation in Hb H/CS disease and the need for further investigation to elucidate underlying mechanisms and potential therapeutic targets.

Principal Component Analysis (PCA) was used to analyze gene expression data and evaluate sample clustering [24]. Based on the results of PCA, a clear separation is evident between the control and Hb H/CS disease samples. This separation is primarily driven by the expression patterns of genes encoding the molecular chaperones or protein folding proteins, including the *HSP* genes, *HSPA8*, *HSP90AA1*, *HSP90AB1*, *HSPA1A*, and *HSPA1B*, as well as the *CCT* genes such as *CCT2*, *CCT3*, *CCT4*, *CCT5*, *CCT6*, *CCT7,* and *CCT8*. These genes form a cluster that effectively distinguishes between normal and diseased samples.

Heat shock proteins have been reported to play roles in erythropoiesis [25,26,27,28,29,30]. The proteins typically serve as protective factors for the erythroid transcription factor GATA1, shielding it from cleavage. Upregulation of these genes occurs in response to cellular stress, playing critical roles in cell repair, survival, and prevention of protein aggregation. Several HSPs are involved in regulating the maturation and differentiation of red blood cells during erythropoiesis.

The chaperonin containing TCP-1 (CCT), also known as chaperonin containing tailless complex polypeptide 1 (CCT) or tailless complex polypeptide 1 ring complex (TRiC), is an essential eukaryotic molecular chaperone. It plays crucial roles in protein folding, preventing protein aggregation, and maintaining homeostasis. CCT is important for the folding of cytoskeletal proteins, especially actin and tubulin, as well as other newly synthesized proteins, and the refolding of aggregated proteins. It also influences mRNA translation, elongation, and post-translational modifications [31]. CCT and the chaperone protein Hgh1 form a multi-partner complex with eukaryotic elongation factor 2 (eEF2), which is an abundant and essential component of the translation machinery. This interaction is crucial for the proper folding of eEF2, enabling its role in translation extension. Moreover, CCT binds to newly synthesized eukaryotic initiation factor 3 (eIF3b) and promotes the correct folding of eIF3h and eIF3i subunits [31,32].

In Hb H/CS disease, numerous stress factors, particularly those associated with the deposition of inclusion bodies in the cell membrane, lead to the induction of reactive oxygen species and damage to the red cell membrane [8,33,34]. The elevated levels of protein folding machinery in Hb H/CS disease may result from cellular toxicity, stress environments, and possibly in response to abnormal precipitates in the red blood cells. Additionally, delayed differentiation of erythroblasts in Hb H/CS disease may contribute to this elevation, as previous studies have indicated a gradual decrease in heat shock protein levels during erythroblast development, resulting in higher levels in early-stage erythroblasts compared to late-stage ones in healthy individuals [30].

A study by Sriiam et al. showed the upregulation of proteins involved in protein folding pathways, the CCT and Hsp70/Hsp90, in erythroblasts derived from patients with Hb H/CS disease compared to erythroblasts from controls. It was proposed that globin chains, either Hb H (β4) or Hb CS, might be assembled in the CCT. However, the upregulation of CCT and Hsp70/Hsp90 might cause an increasing oxidative status in the cell due to the ATP-dependent activity requirement of these protein folding processes [23]. Additionally, a study by Kaewsakulthong et al. demonstrated a significant increase in HSP70 transcripts expression in both α-thalassemia and β-thalassemia, with higher levels observed in α-thalassemia [22]. The findings are consistent with our experimental results and supports the role of heat shock proteins and CCT in erythropoiesis, particularly in the context of Hb H/CS disease.

## 4. Materials and Methods

### 4.1. Patients

Patients aged 10–40 years old with Hb H/CS disease, molecularly confirmed compound heterozygous Southeast Asian deletion and Hb Constant Spring (*HBA2*:c.427T>C) mutation were recruited. Additionally, healthy adults with normal Hb levels and red blood cell indices, Hb analysis, and negative results for α-thalassemia mutations (Southeast Asian, Thai, 3.7 kb, 4.2 kb deletions, Constant Spring and Pakse mutations) were enrolled. Demographic data and hematological characteristics were collected from patients with hemoglobin H/Constant Spring disease and normal controls. The hematological characteristics comprised hemoglobin (Hb), hematocrit (Hct) levels, and red blood cell indices, including red blood cell count (RBC), mean corpuscular volume (MCV), mean corpuscular hemoglobin (MCH), mean corpuscular hemoglobin concentration (MCHC), and red cell distribution width (RDW). Peripheral blood samples were collected from the participants, with 25 mL obtained from each patient and 100 mL from each control for the isolation of CD34+ hematopoietic stem/progenitor cells (HSPCs) to study erythropoiesis and gene expression.

### 4.2. Ethical Issues and Informed Consent

The study received ethical approval from the Research Ethics Committee of the Faculty of Medicine, Chiang Mai University (Study number: PED-2565-09152). Written informed consent was obtained from all participants prior to their enrollment in the study.

### 4.3. Ex Vivo Erythroid Differentiation of Human CD34+ Hematopoietic Stem Cells

The peripheral blood mononuclear cells (PBMCs) were isolated from peripheral blood using Lymphoprep (Axis-Shield, Oslo, Norway) and gradient centrifugation. CD34+ cells were purified from the PBMCs using CD34 microbead kit with LS Columns according to the manufacturer’s protocol (Miltenyi Biotec, Gladbach, Germany) and underwent erythroid lineage differentiation within a 14-day period using a three-phase liquid culture system, which incorporated the use of three distinct erythroid differentiation culture media. The basal medium’s composition consists of Iscove’s modified Dulbecco medium (Cytiva, South Logan, UT, USA) supplemented with 20% *v*/*v* fetal bovine serum (HyClone, Pasching, Austria), 300 μg/mL holo-transferrin (ProSpec, Ness Ziona, Israel), and 1% *v*/*v* penicillin/streptomycin (Gibco, Grand Island, NY, USA) was prepared as a basal medium. During phase I (days 0–4), CD34+ cells were cultured in the basal medium supplemented with specific factors to stimulate the transition from hematopoietic stem cells to erythroid progenitor cells. This supplementation included 10 ng/mL recombinant human interleukin-3 (IL-3; PeproTech, Rehovot, Israel), 50 ng/mL recombinant human stem cell factor (SCF; PeproTech, Rehovot, Israel), and 2 U/mL erythropoietin (EPO; Janssen-Cilag, Bangkok, Thailand). During phase II (days 4–8), the cells were transferred into the basal medium supplemented with 10 ng/mL of SCF and 2 U/mL of EPO to support the expansion of erythroid cells. Finally, during phase III (days 8–14), the cells were cultured in the basal medium supplemented with 4 U/mL of EPO to facilitate erythroid maturation. The cells were incubated at 37 °C in a 5% CO_2_ environment under 100% humidity.

### 4.4. Cell Proliferation and Viability

To assess the cell number and viability of the erythroid cells, we utilized trypan blue staining, followed by counting the cells using a hemocytometer. Cell counts were performed on days 6, 8, and 10 of the culture to assess cell proliferation. The results were reported as a fold change in proliferation relative to the cell count on day 6.

### 4.5. Cell Differentiation Analysis

To evaluate the progress of erythroid differentiation, we examined erythroid cells on days 8, 10, 12, and 14 of culture. The cells were stained with antibodies targeting erythroid surface markers for this assessment. Specifically, we used phycoerythrin (PE)-conjugated anti-human CD71 antibody (clone CY1G4; Biolegend, San Diego, CA, USA) and allophycocyanin (APC)-conjugated anti-human CD235a (Glycophorin A, GPA) antibody (clone HIR2; Biolegend, San Diego, CA, USA). Gating strategies based on the expression of CD71 and GPA markers were employed to distinguish between different stages of erythroblast development. Specifically, R1 (CD71High/GPAHigh) represented basophilic erythroblasts, R2 (CD71Medium/GPAHigh) represented polychromatic erythroblasts, and R3 (CD71Low/GPAHigh) represented orthochromatic erythroblasts and nucleated erythroid cells. Data analysis was conducted using the FlowJo version 10.3.0 (FlowJo LLC, Ashland, OR, USA) software.

### 4.6. RNA Isolation and Sequencing

In selected patients with Hb H/CS disease and controls, RNA extraction was performed on patient-derived cultured cells with a minimum cell count of 1 × 10^6^ cells on day 10. RNeasy Mini Kit (Qiagen, Hilden, Germany) was used, following the manufacturer’s instructions. RNA sequencing was performed by Macrogen, Korea, using the manufacturer’s reagents and protocol. In brief, the sequencing library was prepared from total RNA samples using the TruSeq Stranded Total RNA with the Ribo-Zero Globin kit (Illumina Catalog No. 20020613, Illumina Inc, San Diego, CA, USA). Library sequencing was carried out on an Illumina NovaSeq 6000 system, generating 100-bp paired-end reads. Each output sample consisted of 80 million paired-end reads.

### 4.7. Gene Expression Analysis and Data Visualization

The RNA sequencing data (FASTQ files) quality was assessed, and the results were reported in FastQC [35]. Additionally, all raw FASTQ files underwent data preprocessing with FASTP for adaptor trimming, quality filtering, and read pruning [36]. Subsequently, SortMeRNA was applied to all filtered FASTQ files to remove rRNA sequences [37]. The processed FASTQ files were mapped to the GRCh38.p14 using HISAT2 version 2.1.0 [38]. The BAM alignment files were then subjected to featureCounts version 2.0.1 for read counting [39]. The read counts were analyzed to obtain normalized transcript abundance values (transcripts per million, TPM) for gene expression analysis [40]. Gene annotation was conducted on the transcripts using the BiomaRt package (version 2.60.1) [41].

A Principal Component Analysis (PCA) utilizing FactoMineR (version 2.11) [42] and Factoextra (version 1.0.7) [43] was conducted to evaluate sample clustering based on the gene expression data of selected genes from all nine samples. Subsequently, a heatmap was generated using the pheatmap package (version 1.0.12), employing Euclidean distance [44], to visualize the gene expression data and to extract a sample clustering dendrogram.

### 4.8. Statistical Analysis

Continuous data are presented as the mean ± standard error of the mean (SEM) or the mean ± standard deviation (SD), while categorical data are expressed as numbers and percentages. Student’s *t*-test was employed to compare continuous data among groups, while repeated measures ANOVA was used for comparisons involving repeated measurements. Categorical data were compared among groups using the Chi-square or Fisher’s exact tests. Statistical analyses were conducted using SPSS Statistics for Windows, version 22.0 (IBM Corporation, Armonk, NY, USA), and a *p*-value < 0.05 was considered statistically significant.

## 5. Conclusions

Erythroid progenitor cells derived from patients with Hb H/CS disease demonstrate increased erythroid proliferation, reduced cell viability, and delayed differentiation. Moreover, elevated expression of the molecular chaperones, heat shock proteins, and CCT subunit proteins, was noted, shedding light on potential underlying pathophysiological mechanisms.

## Figures and Tables

**Table 1:** Demographic data and hematological characteristics of patients with hemoglobin H/Constant Spring disease and normal controls.

	Patients with Hb H/CS Disease (N = 12)	Normal Controls (N = 5)	*p*-Value
Age	19.8 ± 7.8	28.8 ± 6.2	0.038
Male sex	7 (58.3%)	3 (60.0%)	>0.999
Hb (g/dL)	8.5 ± 1.5	14.0 ± 1.5	<0.001
Hct (%)	32.7 ± 5.1	44.3 ± 4.5	0.001
RBC (×10^6^/mm^3^)	4.4 ± 0.9	4.9 ± 0.7	0.351
MCV (fL)	73.1 ± 9.5	91.1 ± 4.4	0.001
MCH (pg)	19.6 ± 1.6	28.8 ± 1.8	<0.001
MCHC (g/dL)	27.0 ± 2.2	31.6 ± 0.9	<0.001
RDW (%)	24.2 ± 4.1	13.3 ± 0.9	<0.001

Abbreviations: Hb, hemoglobin; Hct, hematocrit; RBC, red blood cell; MCV, mean corpuscular volume; MCH, mean corpuscular hemoglobin; MCHC, mean corpuscular hemoglobin concentration; RDW, red cell distribution width.

## Data Availability

The authors will share data and protocols to other investigators upon reasonable request.

## References

[1] Lemmens-Zygulska M., Eigel A., Helbig B., Sanguansermsri T., Horst J., Flatz G. (1996). Prevalence of alpha-thalassemias in northern Thailand. Hum. Genet..

[2] Hundrieser J., Sanguansermsri T., Papp T., Flatz G. (1988). Alpha-thalassemia in northern Thailand. Frequency of deletional types characterized at the DNA level. Hum. Hered..

[3] Laig M., Pape M., Hundrieser J., Flatz G., Sanguansermsri T., Das B.M., Deka R., Yongvanit P., Mularlee N. (1990). The distribution of the Hb constant spring gene in Southeast Asian populations. Hum. Genet..

[4] Fucharoen S., Viprakasit V. (2009). Hb H disease: Clinical course and disease modifiers. Hematol. Am. Soc. Hematol. Educ. Program.

[5] Piel F.B., Weatherall D.J. (2014). The α-thalassemias. N. Engl. J. Med..

[6] Hockham C., Ekwattanakit S., Bhatt S., Penman B.S., Gupta S., Viprakasit V., Piel F.B. (2019). Estimating the burden of α-thalassaemia in Thailand using a comprehensive prevalence database for Southeast Asia. eLife.

[7] Clegg J.B., Weatherall D.J., Milner P.F. (1971). Haemoglobin Constant Spring—A chain termination mutant?. Nature.

[8] Schrier S.L., Bunyaratvej A., Khuhapinant A., Fucharoen S., Aljurf M., Snyder L.M., Keifer C.R., Ma L., Mohandas N. (1997). The unusual pathobiology of hemoglobin constant spring red blood cells. Blood.

[9] Charoenkwan P., Taweephon R., Sae-Tung R., Thanarattanakorn P., Sanguansermsri T. (2005). Molecular and clinical features of Hb H disease in northern Thailand. Hemoglobin.

[10] He S., Zheng C., Meng D., Chen R., Zhang Q., Tian X., Chen S. (2015). Hb H Hydrops Fetalis Syndrome Caused by Association of the − −^SEA^ Deletion and Hb Constant Spring (*HBA2*: C.427T>C) Mutation in a Chinese Family. Hemoglobin.

[11] Luewan S., Charoenkwan P., Sirichotiyakul S., Tongsong T. (2020). Fetal haemoglobin H-Constant Spring disease: A role for intrauterine management. Br. J. Haematol..

[12] Charoenkwan P., Sirichotiyakul S., Chanprapaph P., Tongprasert F., Taweephol R., Sae-Tung R., Sanguansermsri T. (2006). Anemia and hydrops in a fetus with homozygous hemoglobin constant spring. J. Pediatr. Hematol. Oncol..

[13] He Y., Zhao Y., Lou J.W., Liu Y.H., Li D.Z. (2016). Fetal Anemia and Hydrops Fetalis Associated with Homozygous Hb Constant Spring (HBA2: C.427T>C). Hemoglobin.

[14] Komvilaisak P., Komvilaisak R., Jetsrisuparb A., Wiangnon S., Jirapradittha J., Kiatchoosakun P., Fucharoen G. (2018). Fetal anemia causing hydrops fetalis from an alpha-globin variant: Homozygous hemoglobin Constant Spring. J. Pediatr. Hematol. Oncol..

[15] Tang H.S., Xiong Y., Li D.Z. (2023). Fetal Hemoglobin H Hydrops Fetalis: Another Three Case Reports. Hemoglobin.

[16] Sirilert S., Charoenkwan P., Sirichotiyakul S., Tongprasert F., Srisupundit K., Luewan S., Tongsong T. (2019). Prenatal diagnosis and management of homozygous hemoglobin constant spring disease. J. Perinatol..

[17] Taghavifar F., Hamid M., Shariati G. (2019). Gene expression in blood from an individual with β-thalassemia: An RNA sequence analysis. Mol. Genet. Genom. Med..

[18] Fakhr-Eldeen A., Toraih E.A., Fawzy M.S. (2019). Long non-coding RNAs *MALAT1*, *MIAT* and *ANRIL* gene expression profiles in beta-thalassemia patients: A cross-sectional analysis. Hematology.

[19] Zhou G., Zhang H., Lin A., Wu Z., Li T., Zhang X., Chen H., Lu D. (2022). Multi-Omics Analysis in β-Thalassemia Using an *HBB* Gene-Knockout Human Erythroid Progenitor Cell Model. Int. J. Mol. Sci..

[20] Mahmoud H.M., Shoeib A.A., Abd El Ghany S.M., Reda M.M., Ragab I.A. (2015). Study of alpha hemoglobin stabilizing protein expression in patients with β thalassemia and sickle cell anemia and its impact on clinical severity. Blood Cells Mol. Dis..

[21] Forster L., McCooke J., Bellgard M., Joske D., Finlayson J., Ghassemifar R. (2015). Differential gene expression analysis in early and late erythroid progenitor cells in β-thalassaemia. Br. J. Haematol..

[22] Kaewsakulthong W., Suriyun T., Chumchuen S., Anurathapan U., Hongeng S., Fucharoen S., Sripichai O. (2022). In Vitro Study of Ineffective Erythropoiesis in Thalassemia: Diverse Intrinsic Pathophysiological Features of Erythroid Cells Derived from Various Thalassemia Syndromes. J. Clin. Med..

[23] Sriiam S., Leecharoenkiat A., Lithanatudom P., Wannatung T., Svasti S., Fucharoen S., Svasti J., Chokchaichamnankit D., Srisomsap C., Smith D.R. (2012). Proteomic analysis of hemoglobin H-constant spring (Hb H-CS) erythroblasts. Blood Cells Mol. Dis..

[24] Roden J.C., King B.W., Trout D., Mortazavi A., Wold B.J., Hart C.E. (2006). Mining gene expression data by interpreting principal components. BMC Bioinform..

[25] Singh M.K., Yu J. (1984). Accumulation of a heat shock-like protein during differentiation of human erythroid cell line K562. Nature.

[26] Arlet J.B., Ribeil J.A., Guillem F., Negre O., Hazoume A., Marcion G., Beuzard Y., Dussiot M., Moura I.C., Demarest S. (2014). HSP70 sequestration by free alpha-globin promotes ineffective erythropoiesis in beta-thalassaemia. Nature.

[27] Kruta M., Sunshine M.J., Chua B.A., Fu Y., Chawla A., Dillingham C.H., Hidalgo San Jose L., De Jong B., Zhou F.J., Signer R.A. (2021). Hsf1 promotes hematopoietic stem cell fitness and proteostasis in response to ex vivo culture stress and aging. Cell Stem Cell.

[28] Weiss M.J., dos Santos C.O. (2009). Chaperoning erythropoiesis. Blood.

[29] Ribeil J.A., Zermati Y., Vandekerckhove J., Cathelin S., Kersual J., Dussiot M., Coulon S., Moura I.C., Zeuner A., Kirkegaard-Sørensen T. (2007). Hsp70 regulates erythropoiesis by preventing caspase-3-mediated cleavage of GATA-1. Nature.

[30] Mathangasinghe Y., Fauvet B., Jane S.M., Goloubinoff P., Nillegoda N.B. (2021). The Hsp70 chaperone system: Distinct roles in erythrocyte formation and maintenance. Haematologica.

[31] Que Y., Qiu Y., Ding Z., Zhang S., Wei R., Xia J., Lin Y. (2024). The role of molecular chaperone CCT/TRiC in translation elongation: A literature review. Heliyon.

[32] Roobol A., Roobol J., Carden M.J., Smith M.E., Hershey J.W., Bastide A., Knight J.R., Willis A.E., Smales C.M. (2014). The chaperonin CCT interacts with and mediates the correct folding and activity of three subunits of translation initiation factor eIF3: B, i and h. Biochem. J..

[33] Kihm A.J., Kong Y., Hong W., Russell J.E., Rouda S., Adachi K., Simon M.C., Blobel G.A., Weiss M.J. (2002). An abundant erythroid protein that stabilizes free alpha-haemoglobin. Nature.

[34] Bunyaratvej A., Sahaphong S., Bhamarapravati N., Wasi P. (1983). Different patterns of intraerythrocytic inclusion body distribution in the two types of haemoglobin H disease. An Ultrastructural Study. Acta Haematol..

[35] Thrash A., Arick M., Peterson D.G. (2018). Quack: A quality assurance tool for high throughput sequence data. Anal. Biochem..

[36] Chen S., Zhou Y., Chen Y., Gu J. (2018). fastp: An ultra-fast all-in-one FASTQ preprocessor. Bioinformatics.

[37] Kopylova E., Noé L., Touzet H. (2012). SortMeRNA: Fast and accurate filtering of ribosomal RNAs in metatranscriptomic data. Bioinformatics.

[38] Kim D., Paggi J.M., Park C., Bennett C., Salzberg S.L. (2019). Graph-based genome alignment and genotyping with HISAT2 and HISAT-genotype. Nat. Biotechnol..

[39] Liao Y., Smyth G.K., Shi W. (2014). featureCounts: An efficient general purpose program for assigning sequence reads to genomic features. Bioinformatics.

[40] Lataretu M., Hölzer M. (2020). RNAflow: An Effective and Simple RNA-Seq Differential Gene Expression Pipeline Using Nextflow. Genes.

[41] Smedley D., Haider S., Ballester B., Holland R., London D., Thorisson G., Kasprzyk A. (2009). BioMart–biological queries made easy. BMC Genom..

[42] Lê S., Josse J., Husson F. (2008). FactoMineR: An R Package for Multivariate Analysis. J. Stat. Softw..

[43] Kassambara A., Mundt F. Factoextra: Extract and Visualize the Results of Multivariate Data Analyses 2020. https://CRAN.R-project.org/package=factoextra.

[44] Kolde R. (2019). Pheatmap: Pretty Heatmaps. https://CRAN.R-project.org/package=pheatmap.

